# Hydrodynamic‐Assisted Liposuction With Stab‐Flatten Technique Versus Traditional Excision for Gynecomastia: A Retrospective Cohort Study

**DOI:** 10.1111/jocd.71147

**Published:** 2026-08-19

**Authors:** Juan Zhang, Jiaomiao Pei, Chaohua Liu, Jianke Ding, Jiangbo Cui, Bingwen Yan, Ying Ma, Baoqiang Song, Dongyue Hao

**Affiliations:** ^1^ Department of Plastic Surgery, Xijing Hospital Fourth Military Medical University Xi'an China

**Keywords:** gynecomastia, hydrodynamic‐assisted liposuction, minimally invasive surgery

## Abstract

**Background:**

Traditional combined surgery for moderate to severe gynecomastia (Simon Grade IIb–III) often results in significant trauma, visible scarring, and contour irregularity.

**Objective:**

To compare outcomes between traditional liposuction/excision (Group A) and a minimally invasive approach using hydrodynamic‐assisted liposuction with a stab‐flatten technique (Group B).

**Methods:**

A retrospective cohort study analyzed 112 patients (214 breasts) treated between 2023 and 2025. Group A (*n* = 50) underwent traditional liposuction and periareolar excision. Group B (*n* = 62) received the novel minimally invasive technique. Evaluation was based on the Breast Evaluation Questionnaire (BEQ), operative time, and complications.

**Results:**

Group B showed significantly superior BEQ scores for chest appearance, body image, satisfaction, and breast firmness. Operative duration was shorter in Group B. The incidence of postoperative complications, including nipple‐areolar complex depression, hematoma, sensory abnormality, and scarring, was slightly lower in Group B.

**Conclusion:**

The hydrodynamic‐assisted liposuction with stab‐flatten technique is a safe, effective, and minimally invasive option. It is associated with improved breast contour, reduced scarring, and enhances patient‐reported outcomes for moderate to severe gynecomastia.

## 1 Introduction

Gynecomastia (GYN) is a benign proliferative disorder of male breast tissue, typically presenting as unilateral or bilateral progressive breast enlargement or a subareolar mass, with or without pain/tenderness, and occasionally nipple discharge. It is common in neonates, adolescents, and middle‐aged to older men. The primary pathogenesis involves hormonal imbalance within breast tissue or altered receptor response. Literature reports a GYN prevalence of 50%–60% in adolescents and up to 70% in men aged 50–69, accounting for 60%–80% of male breast disorders [1]. Although benign, GYN contradicts typical male secondary sexual characteristics, often leading to social anxiety, depression, and even sexual dysfunction, severely impacting patients' psychological well‐being and motivating strong demand for intervention [2]. Recent studies suggest early intervention (age < 18 years) is beneficial, as young men are highly sensitive to peer perception, and their self‐esteem and confidence are more readily affected; thus, early intervention can address body image concerns and restore confidence [3, 4].

Current GYN treatments include medication, surgery, and psychological therapy [5, 6]. Pharmacological treatment remains controversial, lacking robust data to support widespread use. Surgical options include liposuction, open excision, or a combination. Liposuction is primarily suitable for mild GYN, whereas open excision can address skin redundancy but is characterized by significant scarring and trauma [7]. The combined approach leverages the advantages of both methods, effectively reducing breast volume with good aesthetic outcomes, but suffers from complexity and longer operative times [8]. Our team previously explored minimally invasive treatments for GYN and proposed the stab‐flatten technique for severe cases, achieving favorable aesthetic results [9, 10]. This retrospective study compares the efficacy of traditional liposuction combined with open excision versus the refined technique for moderate to severe GYN, aiming to inform treatment strategy selection.

## 2 Patients and Methods

### 2.1 Patients

This study retrospectively reviewed 112 patients with GYN who underwent surgery at our hospital between January 2023 and January 2025. Patients were divided into two groups based on the surgical technique: traditional periareolar incision combined with liposuction (Group A) and hydrodynamic system‐assisted liposuction with the minimally invasive stab‐flatten technique (Group B). Data collected included age, body mass index (BMI), grade of breast enlargement, surgical method, operative duration, and follow‐up time. The study adhered to the Declaration of Helsinki and was approved by the hospital's ethics committee; informed consent was obtained from all patients. Inclusion criteria: (1) Age > 18 years; (2) Simon's Grade IIb–III; and (3) no prior breast surgery or treatment. Exclusion criteria: (1) History of corticosteroid or sex hormone therapy; (2) history of endocrine‐related diseases; (3) breast malignancy; (4) psychiatric conditions unsuitable for surgery; and (5) follow‐up < 6 months or incomplete data. The manuscript was checked against the Strengthening the Reporting of Observational Studies in Epidemiology (STROBE) checklist.

### 2.2 Classification of Gynecomastia

Based on breast volume increase and presence of redundant skin, GYN was classified into three grades and four subtypes (Simon's Grade) [5]: Grade I: Mild glandular hypertrophy, no skin excess; Grade IIa: Moderate glandular hypertrophy, no skin excess; Grade IIb: Moderate glandular hypertrophy, with skin excess; and Grade III: Severe glandular hypertrophy, with skin excess.

### 2.3 Preoperative Assessment

All patients underwent detailed history taking, physical examination, and laboratory tests (progesterone, prolactin, estrogen, androgen) to rule out secondary GYN causes like hypogonadism, hyperthyroidism, or Klinefelter syndrome. Ultrasonography was performed to differentiate pseudo‐gynecomastia and exclude malignancy.

Preoperatively, a Doppler ultrasound blood flow detector is routinely used to identify the perforators of the internal thoracic artery (located in the 2nd–4th intercostal spaces, 1–2 cm parasternal) and the perforators of the lateral thoracic artery (distributed along the anterior axillary line). The Doppler findings do not alter the incision sites or the boundaries of liposuction; rather, they serve solely as a safety guide during the liposuction procedure.

### 2.4 Surgical Technique

#### 2.4.1 Periareolar Incision + Traditional Liposuction (Group A)

Preoperatively, with the patient standing, the liposuction area was marked with gentian violet, extending from the 2nd to 6th intercostal spaces, laterally to the anterior axillary line, and medially to the parasternal line. Under general anesthesia, in the supine position with abducted arms, routine sterilization and draping were performed. A small incision (0.3–0.5 cm) was made laterally/superiorly in the anterior axillary line after infiltration with 0.05% lidocaine and 1:200 000 epinephrine. Approximately 1300–1600 mL of tumescent solution (500 mL saline containing 10 mL of 2% lidocaine and 0.5 mL of 0.1% adrenaline) was infused subcutaneously until the area became pale with a “peau d'orange” appearance. Liposuction was performed using a 4–6 mm cannula via the same incision in a radial pattern, targeting excess fat around and within the gland. A 1 cm thick layer of subcutaneous fat was preserved. Liposuction ceased when minimal fat was aspirated, the area was smooth, and no “step‐off” deformities were present. A 2–3 cm curved incision (not exceeding half the areolar circumference) was made along the inferior areolar border. The gland was dissected and excised, preserving a 0.5–1.0 cm thick layer of subareolar tissue to maintain nipple‐areola complex (NAC) blood supply. After ensuring hemostasis, a drain was placed and secured. Closure was performed in layers. Postoperatively, compression bandaging using dressing pads and elastic bandages is applied for 2–3 months, with uniform pressure to ensure complete adherence of the subcutaneous tissue to the superficial fascia. Dressing change is performed on postoperative Day 1, and sutures are removed at 6–7 days.

#### 2.4.2 Hydrodynamic‐Assisted Liposuction + Minimally Invasive Stab‐Flatten Technique (Group B)

**FIGURE 1:**
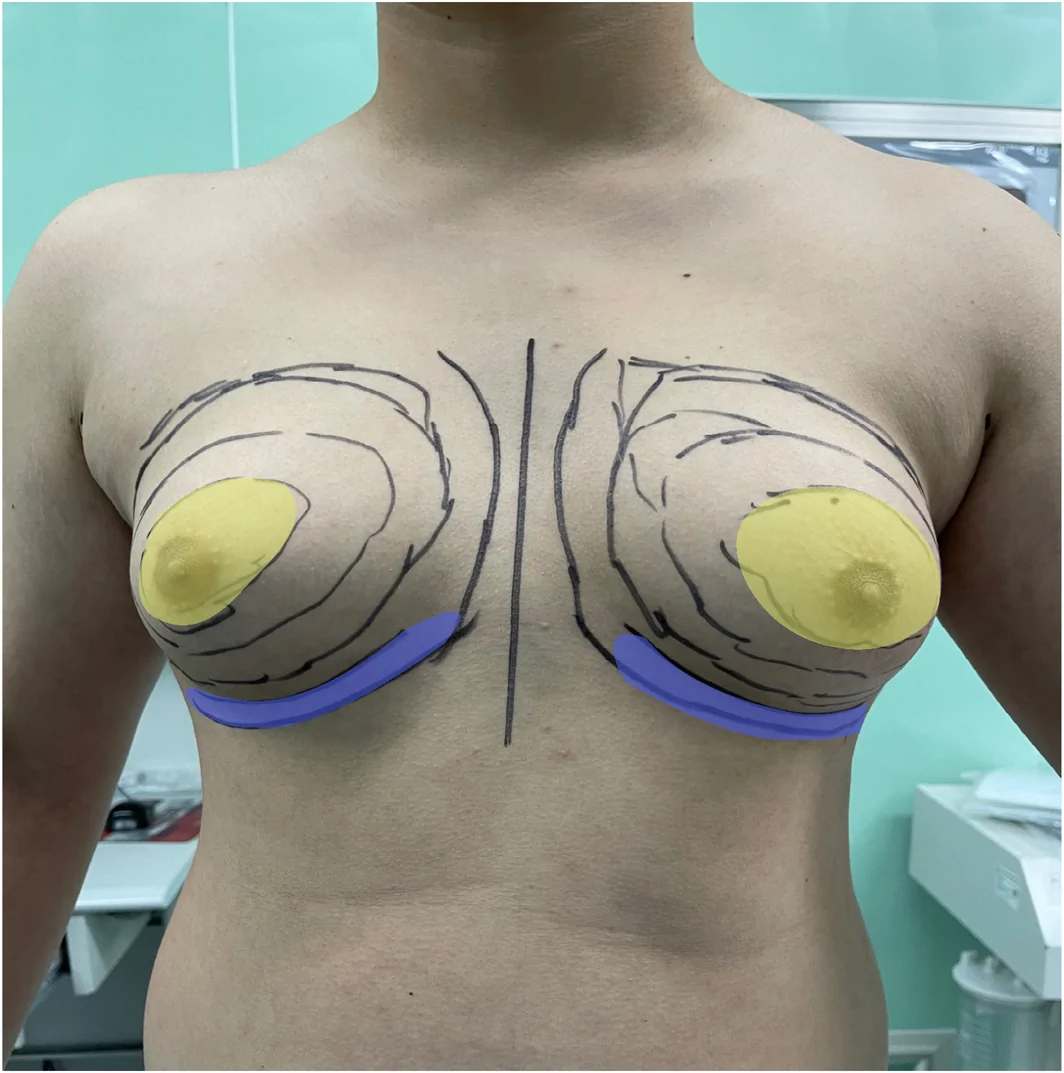
The area outlined by the black circle indicates the liposuction zone for the minimally invasive stab‐flatten technique. The yellow region denotes the key area for focused aspiration and glandular disruption, whereas the blue region represents the liposuction area following release of the inframammary fold ligament. The purpose is to allow better expansion and draping of the loosened skin after liposuction.

**FIGURE 2:**
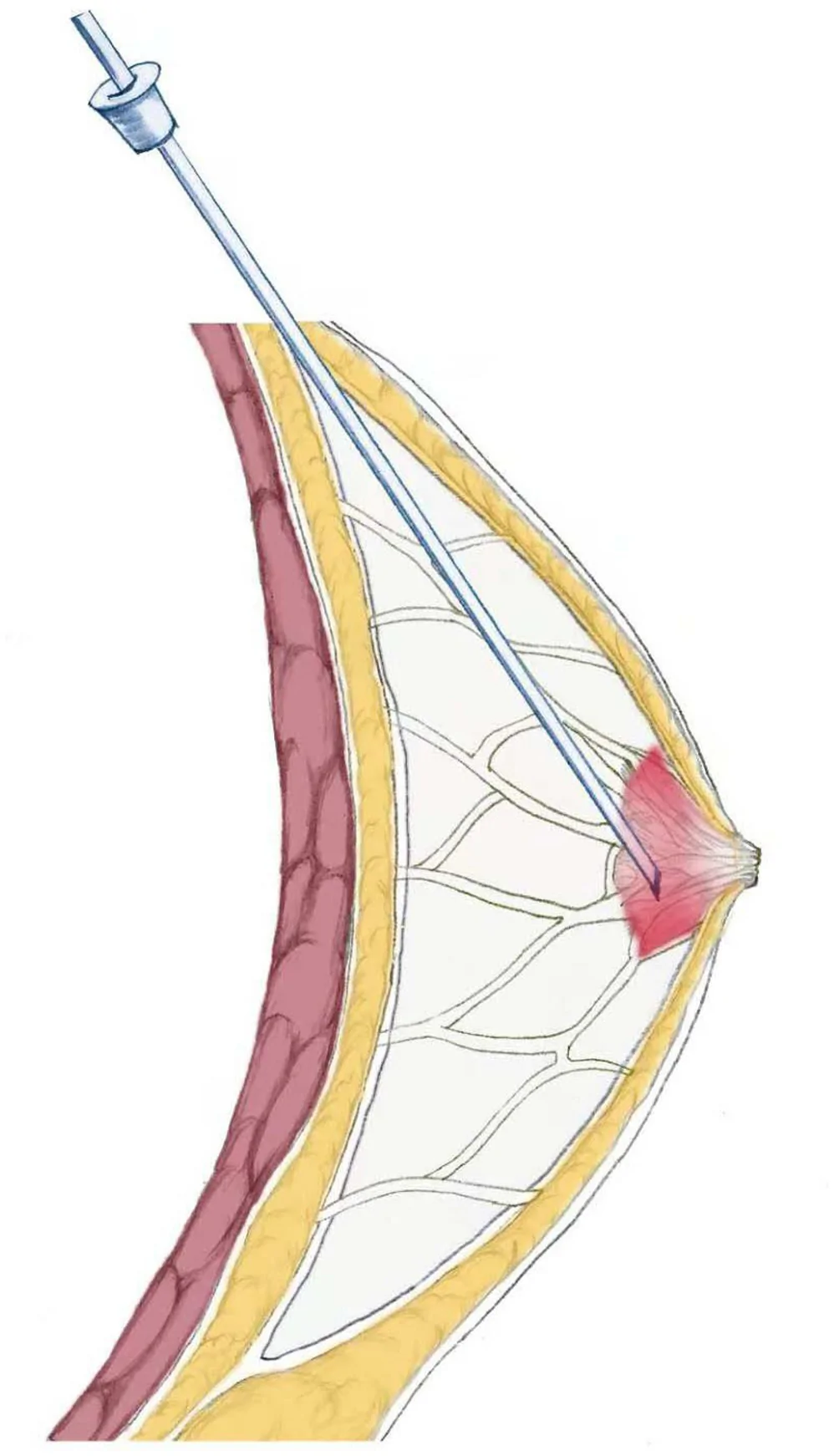
This diagram illustrates the surgical procedure of the minimally invasive stab‐flatten technique.

After anesthesia induction, the patient was positioned supinely. A 0.3–0.4 cm incision was made superolaterally in the anterior axillary line. Tumescent solution (same formula) was injected into the subcutaneous tissue and deep glandular layer. Using the Body‐jet hydrodynamic liposuction system (HUMAN‐MED, Germany), a 2.0 mm diameter water‐jet cannula was used for infiltration (pressure level 1) within the marked area, proceeding from the inferomedial to superolateral quadrants counterclockwise. Infiltration targeted both subglandular (deep to gland) and supraglandular (anterior to gland) planes using the tumescent technique until uniform coverage was achieved. At 55 kPa pressure, a 3.0 mm liposuction cannula was used for repeated aspiration in the plane between the gland and pectoral fascia, and between the gland and skin, preserving a 0.5–1.0 cm subcutaneous fat layer, avoiding over‐suction in the superomedial and superolateral areas (Figure 1). The stab‐flatten technique employed a 35 mm diameter, 30 cm long cannula inserted through the incision. A repeated “stabbing” motion was used to meticulously disrupt the subcutaneous attachments of the breast tissue and the ductal connections to the nipple, until the rib was palpable and the gland felt textureless upon pressure (Figure 2) This technique fragments and redistributes tissue rather than excising it. Care was taken to preserve the subcutaneous glandular tissue beneath the NAC to prevent depression. No drainage tube is required in the incision. The subcutaneous tissue and skin are closed layer by layer. Postoperatively, multiple compression bands are used for 2–3 months, with uniform pressure, to ensure complete adherence of the subcutaneous tissue to the superficial fascia. One band is positioned at the nipple level, and the other bands are superimposed above and below this first band, providing uniform compression and fixation. During the first postoperative month, the compression bands are worn for more than 20 h per day; from the 2nd to the 3rd month, they are worn for approximately 12–18 h per day. Dressing change is performed on postoperative Day 1, and sutures are removed at 6–7 days. Given that the incision is located in a concealed site and its length is only 0.5–1.0 cm, routine anti‐scar gel is not necessary postoperatively.

### 2.5 Surgical Outcome Assessment

Postoperative aesthetic outcomes were evaluated by the surgeon, patient, and a third‐party surgeon using the Breast Evaluation Questionnaire (BEQ) [11]. The BEQ is a 55‐item questionnaire divided into three sections: Part I assesses patient comfort regarding breast size, shape, and firmness in various contexts (intimate, social, professional). Part II evaluates comfort with overall breast appearance in different states of dress (fully clothed, swimsuit, nude), considering situations like being alone, with a partner, with male friends, with familiar females, and with unfamiliar females. Part III contains two questions asking patients to rank satisfaction levels (“Very Dissatisfied” [1] to “Very Satisfied” [5]) from the perspectives of themselves, their spouse/partner, parents, siblings, and friends. If opinions within a category differed, patients selected the opinion most influential to them. All items used a 5‐point Likert scale (1 = Very Dissatisfied; 2 = Dissatisfied; 3 = Neutral; 4 = Satisfied; and 5 = Very Satisfied). To preserve its validated psychometric properties, the questionnaire was used without modification, and participants were instructed to interpret items related to the “breasts” as referring to the chest region.

To evaluate surgical outcomes, the operative time, blood loss, and incidence of postoperative complications were recorded for both surgical techniques. Seroma was defined as a clinically detectable fluid collection confirmed by ultrasound examination on postoperative Day 3; all patients were assessed at each follow‐up visit, and ultrasound was performed if fluid accumulation was suspected. Small seromas were managed with watchful waiting, whereas larger seromas were treated with needle aspiration.

### 2.6 Statistical Analysis

Data were analyzed using SPSS 25.0 (IBM Corp., Armonk, NY, USA). Continuous data are described as mean ± standard deviation. Comparisons between groups were performed using independent samples *t*‐test or Mann–Whitney *U* test. A *p*‐value < 0.05 was considered statistically significant.

## 3 Results

This study included 112 patients (total 214 breasts) with gynecomastia. Group A (traditional liposuction + excision) comprised 50 patients (96 breasts), and Group B (hydrodynamic‐assisted liposuction + stab‐flatten) comprised 62 patients (108 breasts). No significant differences were found in baseline characteristics such as age, BMI, grade of breast enlargement, or follow‐up time between the groups (Table 1). Secondary GYN cases were excluded through preoperative evaluation.

**TABLE 1:** Baseline data.

Variables	Group A	Group B
Patients (breasts)	50 (96)	62 (108)
Age, year	28.56 ± 7.73	27.65 ± 7.56
BMI, kg/m^2^	26.46 ± 1.98	26.18 ± 2.32
Classification		
IIb	32 (60)	38 (73)
III	18 (36)	24 (45)
Follow‐up period, month	10.14 ± 2.76	10.10 ± 2.66

Postoperative aesthetic assessment using the validated BEQ revealed significant differences in selected representative outcomes, summarized in Table 2. Scores for Breast Firmness, General Breast Appearance when nude, General Body Appearance, and Patient Satisfaction were significantly lower in Group A compared to Group B. No significant differences were observed in other assessed items.

**TABLE 2:** Breast evaluation questionnaire score.

Variables	Group A	Group B	*p*	95% CI	Cohen's *d*
Size of breast	4.50 ± 0.51	4.56 ± 0.50	0.50	(−0.26, 0.14)	−0.12
Shape of breast	4.52 ± 0.50	4.68 ± 0.47	0.09	(−0.35, 0.03)	−0.33
Firmness of breast	4.14 ± 0.80	4.42 ± 0.50	0.03*	(−0.53, −0.03)	−0.43
General breast appearance					
Fully dressed	4.48 ± 0.51	4.40 ± 0.50	0.42	(−0.11, 0.27)	0.16
Bathing suit	4.32 ± 0.47	4.50 ± 0.50	0.06	(−0.37, 0.01)	−0.37
Naked	4.14 ± 0.78	4.51 ± 0.50	0.03*	(−0.62, −0.12)	−0.54
General body appearance					
Fully dressed	4.44 ± 0.50	4.55 ± 0.50	0.26	(−0.30, 0.08)	−0.22
Bathing suit	4.51 ± 0.50	4.50 ± 0.50	0.84	(−0.18, 0.20)	0.02
Naked	3.96 ± 0.86	4.48 ± 0.88	0.00*	(−0.83, −0.21)	−0.60
Satisfaction	3.87 ± 0.88	4.58 ± 0.50	0.00*	(−0.99, −0.43)	−1.03

Abbreviation: CI, confidence intervals.

* *p* < 0.05.

Group B had a significantly shorter operative time than Group A, whereas no significant difference was observed in aspirated fat volume. The overall incidence of common complications did not differ significantly between the two groups, although Group B showed slightly lower rates of NAC depression deformity, hematoma, seroma, paresthesia, and scarring (Table 3).

**TABLE 3:** Comparison of operative procedures and postoperative complications.

Variables	Group A (*n* = 50)	Group B (*n* = 62)	*p*	95% CI	Effect sizes
Surgical duration, min	78.80 ± 6.49	65.80 ± 9.17	0.00*	(9.78, 16.22)	1.57
Fat tissue remove, mL	314.50 ± 88.58	335.34 ± 82.19	0.20	(−52.24, 10.56)	−0.25
Complications					
Asymmetry	2	1	0.59	(0.22, 28.64)	2.52
Undercorrection	3	3	1.00	(0.24, 6.31)	1.23
Sunken deformity	5	2	0.24	(0.48, 13.90)	2.59
Hematoma	5	2	0.24	(0.48, 13.90)	2.59
Seroma	3	1	0.47	(0.78, 76.83)	7.75
Paresthesia	4	1	0.19	(0.57, 48.75)	5.27
Scar	5	2	0.24	(0.48, 13.90)	2.59
Infection	2	1	0.59	(0.22, 28.64)	2.52

*Note:* Surgical indicators were compared using independent samples *t*‐test, whereas postoperative complications were analyzed with Fisher's exact test; Cohen's *d* for continuous variables; odds ratios for binary outcomes.

Abbreviation: CI, confidence intervals.

* *p* < 0.05.

## 4 Cases

**FIGURE 3:**
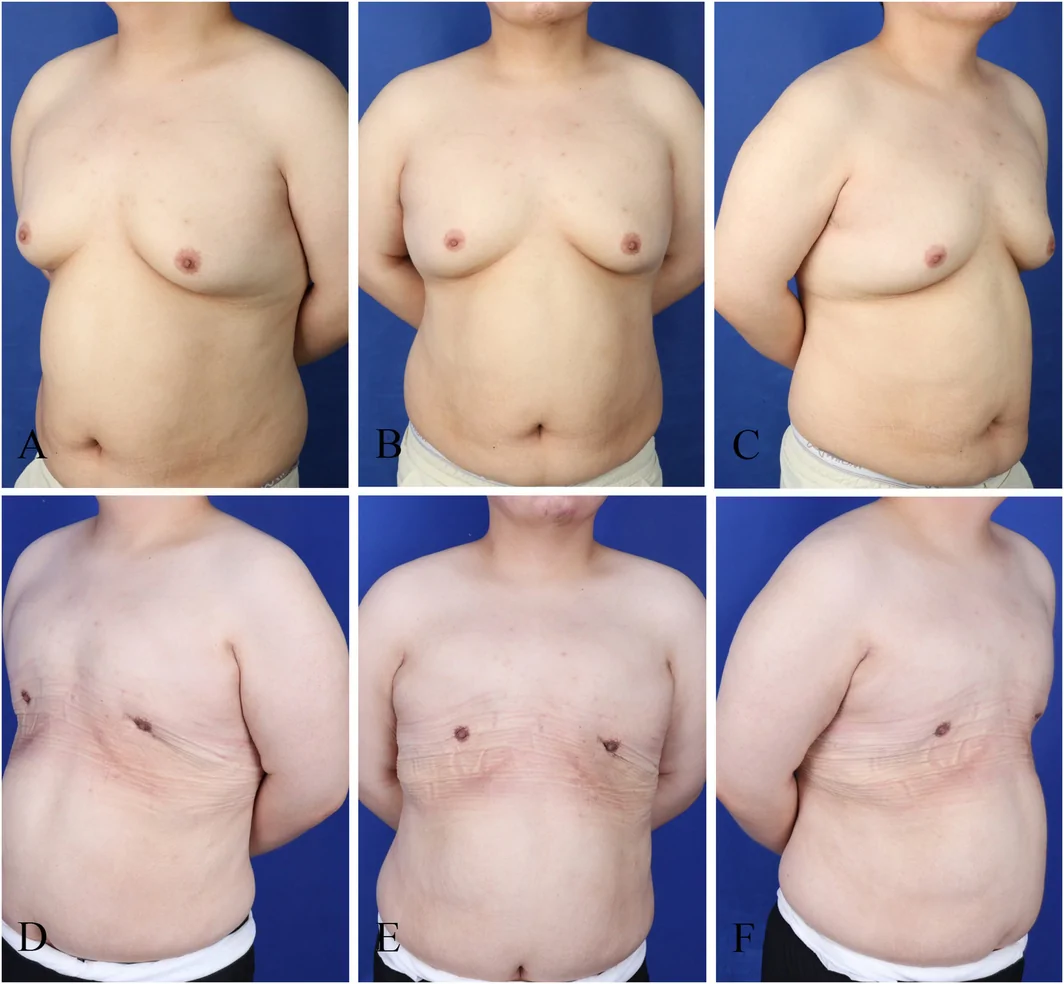
Panels A–C show preoperative photographs of this patient with gynecomastia (GYN, Grade III). Panels D–F show photographs taken 6 months postoperatively.

**FIGURE 4:**
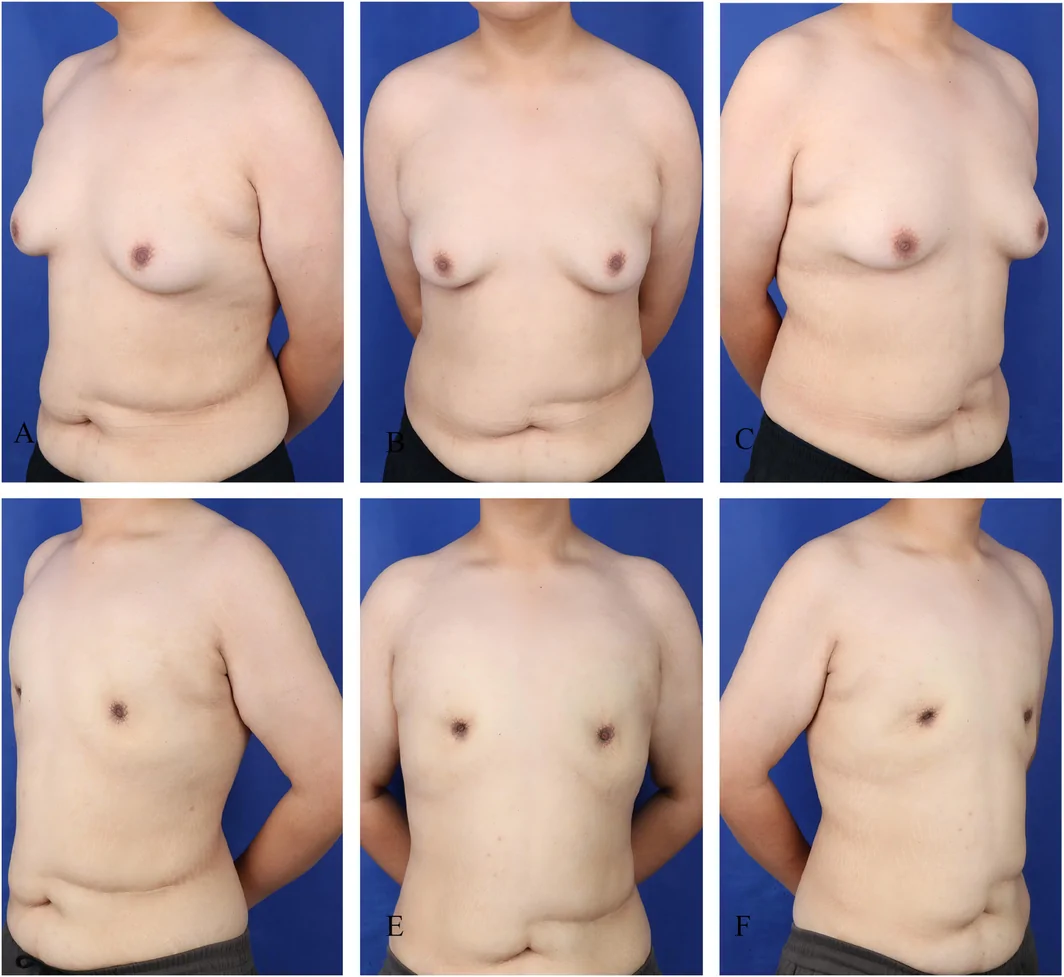
Panels A–C show preoperative photographs of this patient with gynecomastia (GYN, Grade IIb). Panels D–F show photographs taken 6 months postoperatively.

Two male patients with severe breast hypertrophy and excessive skin (Simon Grade III and IIb, respectively) underwent water‐jet assisted liposuction combined with the stab‐flatten technique under general anesthesia, with 350 and 270 mL of adipose tissue aspirated. Both patients had satisfactory recovery and high satisfaction with breast contour at the 6‐month follow‐up (Figures 3 and 4).

## 5 Discussion

The etiology of GYN remains unclear, broadly categorized into physiological glandular overgrowth and pathological hyperplasia. The goals of surgical treatment are to remove hyperplastic tissue causing breast enlargement and reconstruct a normal male chest contour. Surgical indications include: (1) Post‐adolescent males or those past puberty with persistent breast development, where symptoms persist > 1 year after addressing potential causes, and gland diameter > 4 cm; (2) Persistent symptoms > 1 year refractory to medical therapy; (3) Patients with significant psychological burden due to social pressure or breast pain; and (4) Suspected malignancy [6, 12]. Surgical techniques primarily include liposuction, open excision, or combined approaches. Liposuction is mainly for mild–moderate GYN, whereas gland excision suits moderate–severe or predominantly glandular GYN. For severe hypertrophy with skin redundancy, periareolar or extended incisions can remove glandular tissue and excess skin. Common techniques include the double‐circle, omega, and minimally invasive vacuum‐assisted methods. Although open surgery allows thorough removal of hyperplastic tissue and skin, it carries disadvantages like large incisions, significant bleeding, higher infection risk, and prominent scarring [13]. Currently, the combined liposuction and excision technique is widely used for moderate–severe GYN [8, 14]. However, this combined approach also has drawbacks, including suboptimal surgical field exposure, insufficient tissue removal, difficult intraoperative hemostasis, and suboptimal postoperative breast shape. Therefore, key challenges in GYN surgery are the minimally invasive removal of hyperplastic tissue, restoration of male chest contour, and minimization of complications.

The stab‐flatten technique used in this study fragments the hyperplastic glandular tissue and redistributes it around the NAC, effectively addressing poor postoperative contour and step‐off deformities. By preserving the thickness and supporting structures of the NAC, the method avoids nipple necrosis, depression, and sensory abnormalities. By disrupting the rigid fibrous septa of the breast tissue and performing extensive chest wall liposuction, it allows redistribution of lax chest skin without large excisional incisions. The technique utilizes a small, concealed axillary incision, alleviating scar concerns for patients with skin redundancy. Preoperative Doppler mapping of internal thoracic and lateral thoracic artery perforators helps avoid vascular injury during aspiration and reduces hematoma risk. The mechanisms underlying satisfactory skin contraction and redistribution in patients with Simon Grade III include: (1) disruption of the fibrous septa tethering the gland to the dermis; (2) volume reduction through liposuction; (3) preservation of the subareolar tissue to support the nipple–areola complex (NAC); and (4) prolonged compression to facilitate redraping.

Our retrospective comparative study found that hydrodynamic system‐assisted liposuction combined with the stab‐flatten technique significantly reduces breast hyperplasia and reshapes breast contour in moderate–severe GYN, yielding superior breast flatness compared to the traditional combined method. The minimal scarring addresses patient concerns, resulting in significantly better ratings for overall body and chest appearance when nude compared to the traditional group. Furthermore, patients reported higher satisfaction scores with the minimally invasive approach. The stab‐flatten technique also had a shorter operative time compared to the traditional combined surgery.

Compared with our previous study, the present study achieved three substantial advances: (1) introduction of water‐jet assisted liposuction; (2) adoption of a comparative design using conventional suction lipectomy as the control; and (3) inclusion of a larger sample (112 patients, 214 breasts) with systematic patient‐reported outcome assessment. The limitations of this study are as follows: (1) the retrospective design may introduce bias, and prospective randomized controlled trials are needed to validate the findings; (2) owing to the retrospective nature, objective anthropometric parameters—such as interareolar distance, nipple‐to‐axilla distance, nipple‐to‐sternal notch distance, nipple position, chest projection, and other standardized measurements—were not incorporated; and (3) the relatively small, single‐center sample limits generalizability, and future multicenter studies are warranted.

## 6 Conclusion

This retrospective study suggests that hydrodynamic system‐assisted liposuction combined with the minimally invasive stab‐flatten technique appears to address glandular hyperplasia, restore male chest contour, and offer advantages including minimal scarring and a flat appearance. It represents a valuable new option for treating moderate to severe gynecomastia.

## Author Contributions

J.Z., J.P., and C.L. performed the research. C.L. and D.H. supervised the research study. J.D., Y.M., and J.C. analyzed the data. J.Z. and B.Y. wrote the paper. All authors have read and approved the final manuscript.

## Funding

The authors have nothing to report.

## Ethics Statement

All procedures performed in studies involving human participants were in accordance with the ethical standards of the institutional and/or national research committee and with the 1964 Helsinki declaration and its later amendments or comparable ethical standards.

## Conflicts of Interest

The authors declare no conflicts of interest.

## Data Availability

The data that support the findings of this study are available from the corresponding author upon reasonable request.

## References

[1] J. Daniels , A. Brickstock , and R. Charlton , “Gynaecomastia,” BMJ 379 (2022): e069771. 36265883 10.1136/bmj-2021-069771

[2] D. L. Ordaz and J. K. Thompson , “Gynecomastia and Psychological Functioning: A Review of the Literature,” Body Image 15 (2015): 141–148. 26408934 10.1016/j.bodyim.2015.08.004

[3] R. T. Baran , G. Çelmeli , C. Tıkız , A. Önder , and Ö. G. Çoban , “Gynecomastia in Adolescents: Impact on Mental Health and Body Image,” Indian Pediatrics 62 (2025): 521–524. 40531305 10.1007/s13312-025-00113-6

[4] C. T. McNamara , L. C. Nuzzi , J. M. Firriolo , et al., “Complications and Quality of Life Following Gynecomastia Correction in Adolescents and Young Men,” Plastic and Reconstructive Surgery 149 (2022): 1062e–1070e. 10.1097/PRS.0000000000009089 35349529

[5] B. E. Simon , S. Hoffman , and S. Kahn , “Classification and Surgical Correction of Gynecomastia,” Plastic and Reconstructive Surgery 51 (1973): 48–52. 4687568 10.1097/00006534-197301000-00009

[6] A. Cordova and F. Moschella , “Algorithm for Clinical Evaluation and Surgical Treatment of Gynaecomastia,” Journal of Plastic, Reconstructive & Aesthetic Surgery 61 (2008): 41–49. 10.1016/j.bjps.2007.09.033 17983883

[7] A. Innocenti , D. Melita , and E. Dreassi , “Incidence of Complications for Different Approaches in Gynecomastia Correction: A Systematic Review of the Literature,” Aesthetic Plastic Surgery 46 (2022): 1025–1041. 35138423 10.1007/s00266-022-02782-1 PMC9411245

[8] D. H. Kim , I. H. Byun , W. J. Lee , D. K. Rah , J. Y. Kim , and D. W. Lee , “Surgical Management of Gynecomastia: Subcutaneous Mastectomy and Liposuction,” Aesthetic Plastic Surgery 40 (2016): 877–884. 27679453 10.1007/s00266-016-0705-y

[9] B. Yan , D. Hao , L. Sun , et al., “Improved Gynecomastia Surgery: Power‐Assisted Liposuction With Stab‐Flatten Technique Without Resection,” Surgical Innovation 32 (2025): 118–126. 39652398 10.1177/15533506241307270

[10] B. Yan , X. Hong , D. Hao , et al., “Exclusive Liposuction With Glandular Tissue Redistribution for Severe Gynecomastia: A Case Report,” Medicine (Baltimore) 104 (2025): e41299. 39833036 10.1097/MD.0000000000041299 PMC11749746

[11] R. C. Anderson , B. Cunningham , E. Tafesse , and W. R. Lenderking , “Validation of the Breast Evaluation Questionnaire for Use With Breast Surgery Patients,” Plastic and Reconstructive Surgery 118 (2006): 597–602. 16932165 10.1097/01.prs.0000233040.82665.15

[12] G. A. Kanakis , L. Nordkap , A. K. Bang , et al., “EAA Clinical Practice Guidelines‐Gynecomastia Evaluation and Management,” Andrology 7 (2019): 778–793. 31099174 10.1111/andr.12636

[13] T. O. H. Prasetyono , I. Andromeda , and A. G. Budhipramono , “Approach to Gynecomastia and Pseudogynecomastia Surgical Techniques and Its Outcome: A Systematic Review,” Journal of Plastic, Reconstructive & Aesthetic Surgery 75 (2022): 1704–1728. 10.1016/j.bjps.2022.02.008 35304857

[14] W. Xing , C. Liu , L. Chen , Z. Li , and J. Zheng , “Improving Surgical Treatment for Severe Gynecomastia and Avoiding Skin Resection: Skin Redistribution and Fixation Approach for Aesthetic Excellence,” Aesthetic Surgery Journal 46 (2025): 260–268. 10.1093/asj/sjaf193 41002237

